# Association of the IRAK4 rs4251545 genetic polymorphism with severity of enterovirus‐71 infection in Chinese children

**DOI:** 10.1002/iid3.614

**Published:** 2022-04-19

**Authors:** Jie Song, Yedan Liu, Ya Guo, Peipei Liu, Fei Li, Chengqing Yang, Xiaoyu Pan, Liping Yi, Fan Fan, Han Zhao, Zongbo Chen

**Affiliations:** ^1^ Department of Pediatrics The Affiliated Hospital of Qingdao University Qingdao Shandong China; ^2^ Department of Pediatrics Shandong Provincial Hospital Affiliated to Shandong First Medical University Jinan Shandong China; ^3^ Department of Pharmacology and Toxicology University of Mississippi Medical Center Jackson Mississippi USA; ^4^ Department of Pathology The Affiliated Hospital of Qingdao University Qingdao Shandong China

**Keywords:** EV71 infection, IL‐6/NF‐κB, IRAK4, single‐nucleotide polymorphism

## Abstract

**Introduction:**

This study aimed to explore the association between the *IRAK4* polymorphism rs4251545 and the severity of enterovirus 71 (EV71) infection in Chinese children.

**Methods:**

We analyzed the *IRAK4* polymorphism rs4251545 in 617 EV71‐infected patients and 410 controls using the improved multiplex ligation detection reaction. *IRAK4* mRNA expression was tested by qRT‐PCR. Serum concentrations of IL‐6 and NF‐κB were detected using ELISA.

**Results:**

The frequencies of the GA + AA genotype and A allele in the mild EV71 infection group and in the severe EV71 infection group were significantly higher than those in the normal control group. The frequency of the GA + AA genotype and A allele in severely infected EV71 patients was markedly higher than that in mildly infected EV71 patients. *IRAK4* mRNA expression in mildly infected EV71 patients and severely infected patients was significantly higher than that in the control group. *IRAK4* mRNA expression in GA + AA genotypes in both mild and severe EV71 infection groups was significantly higher than that in patients with the GG genotype. IL‐6 concentration and the ratio of IL‐6/NF‐κB in severe EV71 cases were significantly lower in patients with the GA + AA genotype than in those with the GG genotype. The ratio of IL‐6/NF‐κB was distinctly higher in severely infected EV71 patients than in mildly infected and control subjects.

**Conclusions:**

The *IRAK4* polymorphism rs4251545 was associated with the susceptibility and severity of EV71 infection. The A allele is a susceptible factor in the development of severe EV71 infection in Chinese children.

## Graphical abstract

The polymorphism of IRAK4 rs4251545 was associated with the susceptibility and severity of EV71 infection. A allele was a susceptible factor in developing severe EV71 infection in Chinese children.

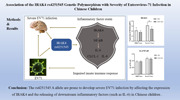

## 1 INTRODUCTION

Enterovirus 71 (EV71), a single‐positive‐sense‐strand neurovirulent RNA virus, is a well‐known causative pathogen of hand−foot−mouth disease (HFMD).^1^, ^2^ Most children infected with EV71 only manifest mild symptoms, such as oral herpes and fever, while a few have severe symptoms, such as neurogenic pulmonary edema, pulmonary hemorrhage, brainstem encephalitis, and circulatory failure.^3^ Since it was first isolated in California in 1969 and identified as the causative agent of the HFMD epidemic in Japan in 1973, EV71 infections have continued to be reported, especially in a large‐scale Asia Pacific pandemic in 2008 in China. In that outbreak, nearly 490,000 children were infected with EV71, including 126 patients who died.^4^ Although EV71 vaccine research has made breakthrough progress, no effective antiviral drugs have been developed for EV71 infection to date. Therefore, it is still significant to explore the risk factors of EV71 infection, especially severe infection, and to develop effective prevention and treatment measures.^5^, ^6^

SInnate immunity is the first barrier against EV71 infection. The interleukin 1 receptor‐associated kinase (IRAK) family plays a crucial role in the innate immune system by participating in signaling networks of the innate axis of the immune response as an intracellular kinase. These networks are important for the antiviral response, regulation of inflammation, and control of autoimmune and inflammatory diseases.^7^ *IRAK4*, a member of the IL1R family, is an important mediator of toll‐like receptor signal transduction. *IRAK4* contains an N‐terminal DD, a pro‐ST domain, and a central conserved kinase domain.^8^, ^9^ The DD domain is vital for signaling transmission because of its interaction with signaling molecules, such as MyD88.^10^, ^11^ IRAK4 reportedly mediates NF‐κB activation by affecting the transmission of information between TIR and MyD88, thus causing the release of downstream inflammatory factors.^12^ Besides, IL‐6, an important proinflammatory factor, could activate the immune system and affect the viral load after pathogen infection. The human *IRAK4* gene, located on chromosome 12q12, contains 12 exons and encodes a protein of 460 amino acids and shares 87% similarity and 84% identity with murine *Irak4*.^13^ It is widely known that the host genetic background is related to the occurrence and development of diseases. Studies have shown that the *IRAK4* polymorphism rs4251545 is related to several diseases, such as severe sepsis, hepatocellular carcinoma (HCC), and breast cancer.^14^, ^15^, ^16^ Rs4251545 changes the G allele to the A allele in the 11th exon of *IRAK4*, which causes an alanine‐to‐threonine substitution at amino acid 428 in the *IRAK4* protein. Susan^16^ indicated that this missense mutation introduces a potential phosphorylation site in the extreme carboxy terminus (XCT) of the *IRAK4* kinase domain, and that the XCT subdomain of *IRAK4* performs biological functions in breast cancer. However, the relationship between rs4251545 and EV71 infection remains unclear.

This study explored the relationship between the *IRAK4* polymorphism rs4251545 and EV71 infection in Chinese children to determine whether this variant of *IRAK4* is associated with an increased risk of EV71 infection. This will provide novel insights into the mechanism and treatment of EV71 infection.

## 2 METHODS

### 2.1 Case selection

**Figure 1:**
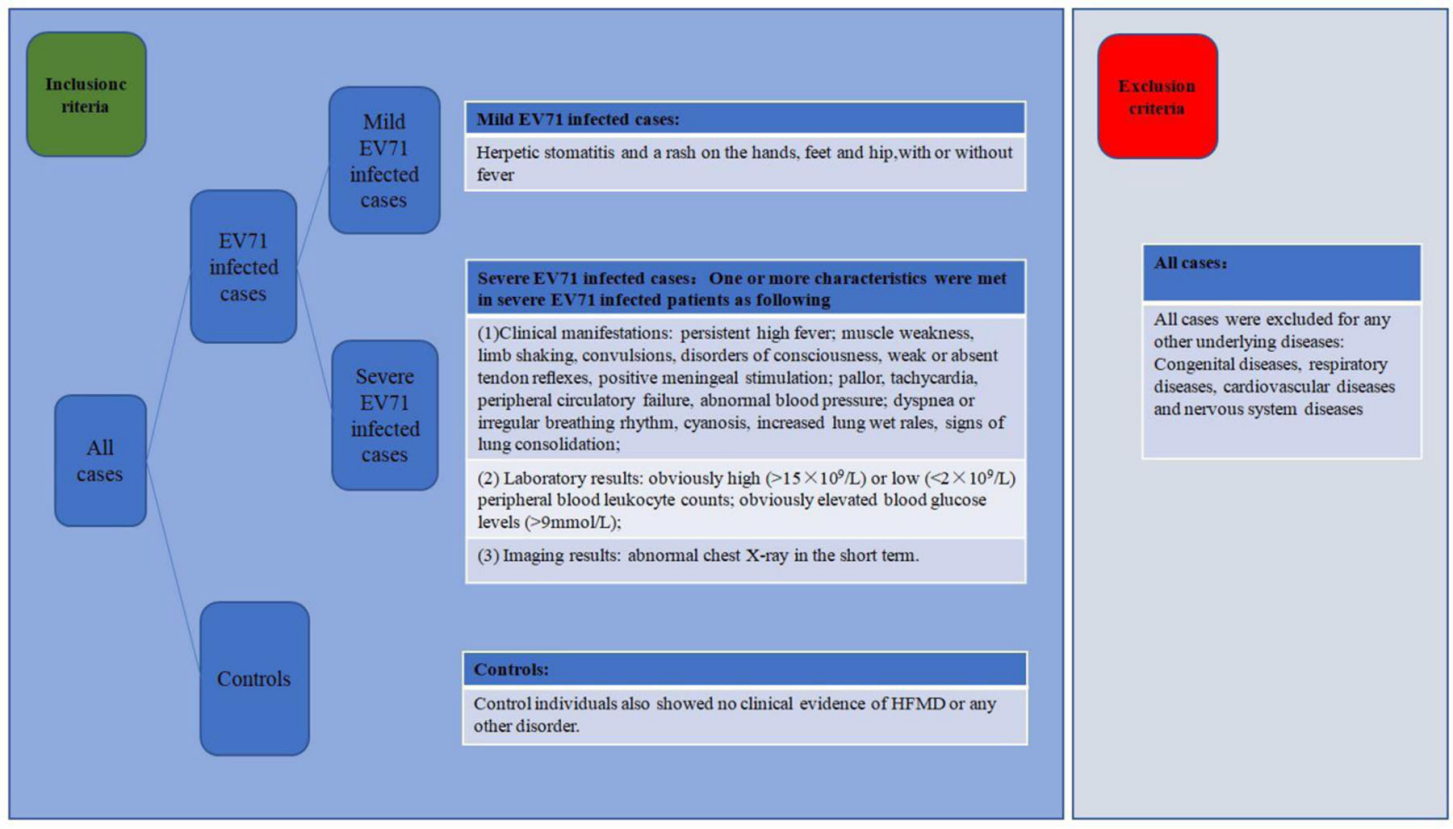
The inclusion/exclusion criteria of this study

We collected data from 410 healthy children undergoing physical examination and 621 EV71‐infected children treated at the Affiliated Hospital of Qingdao University, Qingdao Women & Children's Hospital, and Affiliated Hospital of Jining Medical University between 2013 and 2019; we grouped them according to their clinical diagnostic criteria. Four cases in the infected group were excluded for underlying diseases: congenital heart disease (*n* = 1) and epilepsy (*n* = 3). EV71 infection was confirmed by both clinical features and a reverse transcription polymerase chain reaction (RT‐PCR). RNA was extracted from stool specimens obtained from the patients on the day after admission. Clinical and laboratory data were collected. Inclusion/exclusion criteria are shown in Figure 1. All the procedures in this study were in accordance with the standards of the Ethics Review Committee of the Affiliated Hospital of Qingdao University. We obtained informed consent from the children's parents.

### 2.2 Genomic DNA isolation and genotyping of IRAK4 rs4251545

Genomic DNA was extracted from peripheral blood using a commercial kit (Qiagen). To detect *IRAK4* rs4251545, the iMLDR technique developed by Genesky Biotechnologies Inc. was used for genotyping. The size was 144 bp. The PCR products were purified by digestion with 5 U of shrimp alkaline phosphatase and 2 U exonuclease I at 37°C for 1 h and at 75°C for 15 min.

The ligation reaction mixture contained 1 μl 10× ligase buffer, 0.25 μl high‐temperature ligase, 0.4 μl 5′ primer mixture (1 μm), 0.4 μl 3′ primer mixture (2 μm), 2 μl purified multiplex PCR product, and 6 μl ddH_2_O. In a double connection reaction, each site contained two 5′ ends of allele‐specific probes, followed by a 3′ end of an allele‐specific probe. Each allele‐specific connection product was distinguished by its fluorescence, while different loci were distinguished by the different lengths added to the 3′ end of the allele‐specific probe.

They were genotyped using an ABI3730XL Genetic Analyser, and the raw data were validated using GeneMapper 4.1 (Applied Biosystems). The specific primers and cycling programs are listed in Table 1.

**Table 1:** Primers and PCR conditions

Item		Sequence (5'−3')	The PCR conditions
IRAK4 rs4251545	Forward primer	GATGAATGATGCTGATTCCACTTCAG	95°C for 2 min; 11 cycles at 94°C for 20 s, 65–0.5°C/cycle for 40 s, and 72°C for 1 min 30 s; and 24 cycles at 94°C for 20 s, 59°C for 30 s, and 72°C for 1 min 30 s, followed by 72°C for 2 min and holding at 4°C.
	Reverse primer	TGAACCCCACCCCTTTCACTTT	
IRAK4 rs4251545 probes	FA	TGTTCGTGGGCCGGATTAGTAATGATGCTGATTCCACTTCAGTTGCAA	38 cycles at 94°C for 1 min and 56°C for 4 min, and then holding at 4°C.
	FG	TCTCTCGGGTCAATTCGTCCTTAATGATGCTGATTCCACTTCAGTTGTAG	
	FP	CTATGTACTCTGTTGCTAGTCAATGTCTGCTTTTTTT.	
IRAK4	Forward primer	TGATGGAGATGACCTCTGCT	95°C for 30 s, followed by 40 cycles of 95°C for 10 s, and 60°C for 30 s.
	Reverse primer:	GGTGGAGTACCATCCAAGCAA	
β‐actin	Forward primer	AAGAGAGGCACCTCACCCT	
	Reverse primer:	GGAAGGAAGGCTGGAAG	
EV71 VP1	EV71‐S	GTTCTTAACTCACATAGCA	
	EV71‐A	TTGCAAAAACTGAGGGTT	

### 2.3 RNA isolation and reverse transcription‐quantitative PCR (RT‐qPCR)

TRIzol reagent (Invitrogen) was used to purify total RNA from peripheral blood lymphocytes in accordance with the manufacturer's protocol. Peripheral blood was collected on the second day after admission, and peripheral blood lymphocytes were extracted according to the manufacturer's instructions (Solarbio). A QuickDrop spectrophotometer was used to assess the concentration and quality of the RNA. Each sample was reverse‐transcribed into cDNA and analyzed using the SYBR‑Green Real‑Time PCR kit (Vazyme). The $2^{‐∆∆C_{q}}$ method was used for the relative quantification of *IRAK4* mRNA. A series of dilutions (1 × 10^7^, 1 × 10^6^, 1 × 10^5^, and 1 × 10^4^ copies/μl of a DNA fragment) derived from EV71 were used to create a standard curve for calculating the copy numbers of viral RNA in various samples. Quantitative RT‐PCR was performed using a Linegene9660 system (Thermo Fisher Scientific). The specific primers and cycling programs are listed in Table 1.

### 2.4 Estimation of NF‐κB and IL‐6 levels

Serum NF‐κB and IL‐6 levels were measured in 32 controls, 31 mild EV71 cases, and 39 severe EV71 cases. Plasma concentrations of NF‐κB and IL‐6 were detected using ELISA kits (Elabscience) according to the manufacturer's instructions. The sensitivities of NF‐κB and IL‐6 level detection were 100 and 4.69 pg/ml, respectively, as indicated by the manufacturer. Each sample was assessed three times, and the values were in the linear part of the standard curve.

### 2.5 Statistical analysis

The genotypes were tested for Hardy–Weinberg equilibrium using the *χ* ^2^ test. The *χ* ^2^ test was used to compare the frequencies of genotypes and alleles in the different groups. The relationship between *IRAK4* rs4251545 and the susceptibility and severity of EV71 infection and encephalitis was evaluated by calculating the odds ratio (OR) and 95% confidence intervals (95% CI) using logistic regression. Parametric data are presented as mean ± standard deviation (SD) and were analyzed using the *t* test. Nonparametric data were analyzed using the Kruskal−Wallis test and are presented as median values (25th–75th percentage values). All statistical analyses were performed using SPSS 21.0 and significance was set at *p* < .05.

## 3 RESULTS

### 3.1 Study population

A total of 410 controls (193 males) and 617 EV71‐infected patients (277 males) were examined. There was no significant difference in age (*Z* = 1.119, *p* = .263) and sex ( *χ* ^2^ = 0.471, *p* = .493) between EV71‐infected patients and controls. The EV71‐infected patients were divided into two groups: the severe infection group and the mild infection group. The severe group consisted of 181 patients (82 males), including 78 encephalitis cases (41 males); the mild EV71 infection group consisted of 436 patients (195 males). No significant differences in age (*Z* = 0.815, *p* = .415) or gender ( *χ* ^2^ = 0.017, *p* = .895) were found between patients with severe and mild EV71 infection.

### 3.2 Distribution of genotypes and alleles of IRAK4

The genotype and allele distributions of each group obeyed the Hardy−Weinberg equilibrium (*p* > .05) (Tables 2 and 3). EV71‐infected patients had a significantly higher frequency of the GA + AA genotype (*p* = .001) than did the controls. However, there was no clear discrepancy in the frequency of the A allele (*p* = .059). The frequencies of the GA + AA genotype and A allele in severely infected EV71 patients were higher than those in the controls (*p* = .003 and *p* = .004, respectively). The same result was obtained when comparing severely infected EV71 patients and mildly infected patients (*p* = .046 and *p* = .033, respectively) (Table 4). Furthermore, a similar difference was found between EV71 patients with encephalitis and the controls (*p* = .002 and *p* = .002, respectively) (Table 5).

**Table 2:** IRAK4 rs4251545G/A genotype and allele frequencies in the EV71‐infected group

Item	*n*	Genotype			Allele	
		AA	GA	GG	A	G
Actual frequency	617	3 (0.5%)	95 (15.4%)	519 (84.1%)	101 (8.2%)	1133 (91.8%)
H−W theoretical frequency		4 (0.6%)	93 (15.1%)	520 (84.3%)		
*χ* ^2^			0.369			
*p*			.544			

**Table 3:** IRAK4 rs4251545G/A genotype and allele frequencies in the controls

Item	*n*	Genotype			Allele	
		AA	GA	GG	A	G
Actual frequency	410	3 (0.7%)	43 (10.5%)	364 (88.8%)	49 (6.0%)	771 (94.0%)
H−W theoretical frequency		1 (0.2%)	46 (11.2%)	362 (88.3%)		
*χ* ^2^			1.823			
*p*			.177			

**Table 4:** Genotype and allele frequencies of the IRAK4 rs4251545G/A polymorphism in EV71‐infected patients and controls

IRAK4 rs4251545 G/A	EV71‐infected patients (*n* = 617)	Mild cases (*n* = 436)	Severe cases (*n* = 181)	Controls (*n* = 410)	*χ* ^2^	*p* value	OR (95% CI)
Genotype							
					10.546^a^	.001^a^	1.988 (1.305−3.030)^a^
					1.469^b^	.226^b^	1.287 (0.855−1.938)^b^
GA + AA	98 (15.9%)	61 (14.0%)	37 (20.4%)	46 (11.2%)	8.847^c^	.003^c^	2.033 (1.266−3.266)^c^
GG	519 (84.1%)	375 (86.0%)	144 (79.6%)	364 (88.8%)	3.984^d^	.046^d^	1.580 (1.006−2.481)^d^
Allele							
					3.552^a^	.059^a^	1.403 (0.985−1.997)^a^
					0.887^b^	.346^b^	1.204 (0.818−1.774)^b^
A	101 (8.2%)	62 (7.1%)	39 (10.8%)	49 (6.0%)	8.390^c^	.004^c^	1.900 (1.223−2.950)^c^
G	1133 (91.8%)	810 (92.9%)	323 (89.2%)	771 (94.0%)	4.568^d^	.033^d^	1.577 (1.036−2.403)^d^

Abbreviations: CI, confidence interval; OR, odds ratio.

^a^ EV71‐infected patients versus controls using the *χ* ^2^ test with a 2 × 2 contingency table.

^b^ Mild cases in EV71‐infected patients versus controls using the *χ* ^2^ test with a 2 × 2 contingency table.

^c^ Severe cases versus controls in EV71‐infected patients using the *χ* ^2^ test with a 2 × 2 contingency table.

^d^ Severe versus mild cases in EV71‐infected patients using the *χ* ^2^ test with a 2 × 2 contingency table.

**Table 5:** Genotype and allele frequencies of the IRAK4 rs4251545G/A polymorphism in EV71 encephalitis and nonencephalitis

IRAK4 rs4251545G/A	EV71 encephalitis (*n* = 78)	Nonencephalitis (*n* = 103)	Controls (*n* = 410)	*χ* ^2^	*p* value	OR (95% CI)
Genotype				9.799^a^	.002^a^	2.548 (1.397−4.648)^a^
GA + AA	19 (24.4%)	18 (17.5%)	46 (11.2%)	2.951^b^	.086^b^	1.676 (0.925−3.035)^b^
GG	59 (75.6%)	85 (82.5%)	364 (88.8%)	1.293^c^	.255^c^	0.658 (0.318−1.358)^c^
Allele						
				9.347^a^	.002^a^	2.314 (1.334−3.506)^a^
A	20 (12.8%)	19 (9.2%)	49 (6.0%)	2.806^b^	.094^b^	1.599 (0.919−2.780)^b^
G	136 (87.2%)	187 (90.8%)	771 (94.0%)	1.195^c^	.274^c^	0.691 (0.355−1.344)^c^

Abbreviations: CI, confidence interval; OR, odds ratio.

^a^ EV71 encephalitis patients versus controls using the *χ* ^2^ test with a 2 × 2 contingency table.

^b^ Nonencephalitis patients versus controls using the *χ* ^2^ test with a 2 × 2 contingency table.

^c^ EV71 encephalitis patients versus nonencephalitis patients in EV71‐infected patients using the *χ* ^2^ test with a 2 × 2 contingency table.

### 3.3 Analysis of clinical features

In EV71‐infected cases, patients with the GA + AA genotype had higher white blood cell counts (*p* < .001), blood glucose (BG) (*p* < .001) concentration, and a longer duration of fever (*p* = .049). Lower alanine aminotransferase levels were found in patients with the GA + AA genotype (*p* = .001). The percentages of seizure (*p* < .001), spirit change (*p* = .007), and abnormal electroencephalography (EEG) (*p* = .005) in patients with the GA + AA genotypes were obviously higher than those in the patients with the GG genotypes among EV71‐infected cases. However, there were no significant differences in vomiting and abnormal brain magnetic resonance imaging (MRI) (Table 6).

**Table 6:** Characteristic of EV71‐infected group according to different genotypes

Parameters	GA + AA (*n* = 98)	GG (*n* = 519)	*χ* ^2^/*t*/*t*'/*Z*	*p* value
Male (*n* = 253)	25	228		
Female (*n* = 332)	41	291	0.874	.350^a^
Age (years)	6.3 ± 2.4	6.4 ± 2.2	−0.359	.719^b^
Duration of fever (days)	4.0 (2.0−6.0)	3.0 (1.0−6.0)	1.971	.049^c^
WBC (×10^9^/L)	9.2 ± 1.9	6.6 ± 1.8	12.837	<.001^c^
CRP (mg/L)	14.2 ± 3.8	14.9 ± 3.9	−1.618	.099^c^
ALT (U/L)	22.5 (15.0−33.5)	30.0 (17.0−49.0)	3.442	.001^c^
AST (U/L)	36.5 (21.0−50.3)	37.0 (22.0−39.0)	0.593	.553^c^
CK‐MB (U/L)	20.0 (14.0−29.0)	20.0 (13.0−28.0)	0.855	.393^c^
BG (mmol/L)	9.1 ± 2.2	6.1 ± 1.7	15.243	<.001^c^
Vomiting	30 (30.6%)	154 (29.7%)	0.035	.852^a^
Seizure	27(27.6%)	70 (13.5%)	12.305	<.001^a^
Spirit change	22 (22.4%)	63 (12.1%)	7.377	.007^a^
Brain MRI abnormal	13 (13.3%)	67 (12.9%)	0.009	>.923^a^
EEG abnormal	22 (22.4%)	62 (11.9%)	7.732	.005^a^
EV71 load (log10 copies/μl)	3.92 ± 0.69	4.01 ± 0.70	1.225	.221^b^

Abbreviations: ALT, alanine aminotransferase; AST, aspartate aminotransferase; BG, blood glucose; CK‐MB, cardiac creatine kinase‐MB fraction; CRP, C‐reactive protein; EEG, electroencephalography; MRI, magnetic resonance imaging; WBC, white blood cell count.

^a^ Groups compared using *χ* ^2^ test.

^b^ Groups compared using the *t* test, values expressed as mean ± SD.

^c^ Groups compared using the Wilcoxon rank‐sum test, values expressed as median (25th–75th percentile values).

### 3.4 Quantification of IRAK4 mRNA

**Figure 2:**
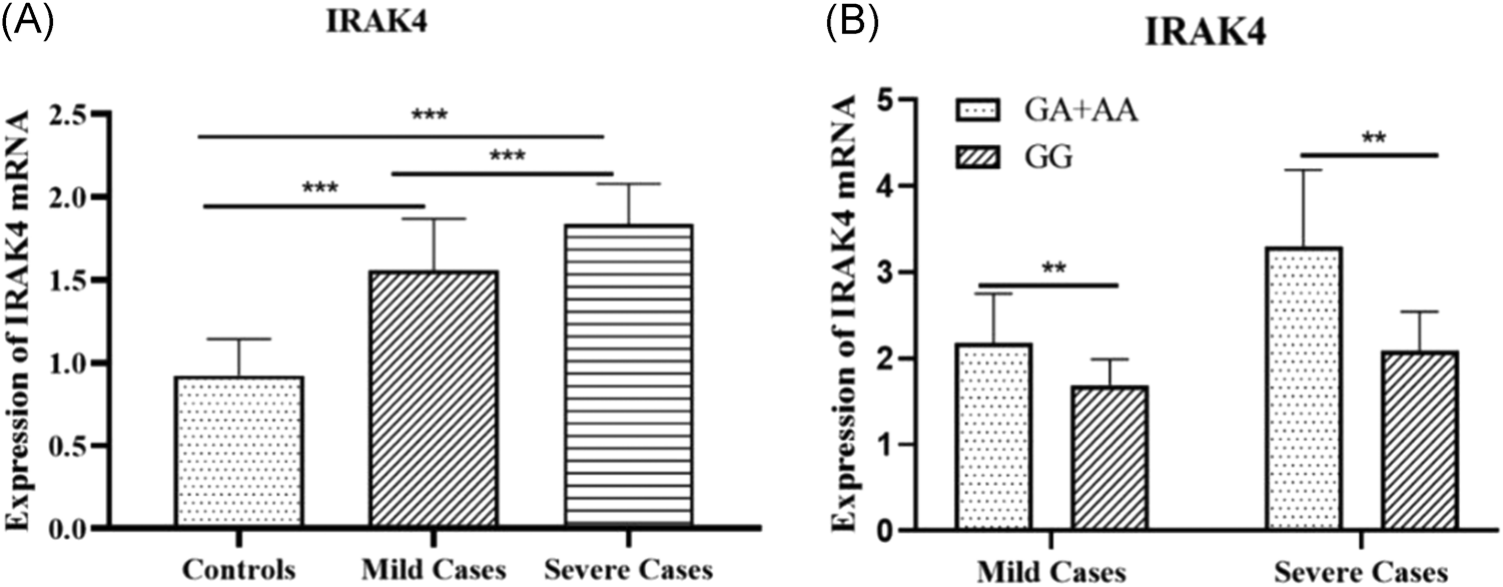
Expression of IRAK4 mRNA in peripheral blood lymphocytes were detected in 32 controls, 31 mild EV71‐infected patients, 39 severe EV71‐infected patients. Values expressed as mean ± SD. (A) Expression of IRAK4 mRNA in mild EV71‐infected patients and severe cases were significantly higher than in controls (*p *< .001 and *p* < .001, respectively). The similar difference was found between mild and severe EV71‐infected cases (*p* < .001). (B) Expression of IRAK4 mRNA in GA + AA genotypes was significantly higher than in GG genotypes patients both in severe and mild EV71 patients (*p* < .01 and *p* < .01, respectively). **p < .01, ***p < .001

As shown in Figure 2, the expression of *IRAK4* mRNA in severely infected EV71 patients (1.8 ± 0.2) and mildly infected patients (1.6 ± 0.3) was significantly higher than that in the controls (0.9 ± 0.2) (*p* < .001 and *p* < .001, respectively). A similar difference was found between the severe and mild EV71 infected cases (*p* < .001). The expression of *IRAK4* mRNA in severely infected EV71 patients with the GA + AA genotype (3.3 ± 0.9) was significantly higher than that in patients with the GG genotype (2.1 ± 0.5) (*p* < .01). *IRAK4* mRNA in mildly infected EV71 patients with the GA + AA genotype (2.2 ± 0.6) was also significantly higher than in patients with the GG genotype (1.7 ± 0.3) (*p* < .01).

### 3.5 Effects of IRAK4 rs4251545 polymorphism on serum NF‐κB and IL‐6 level

**Figure 3:**
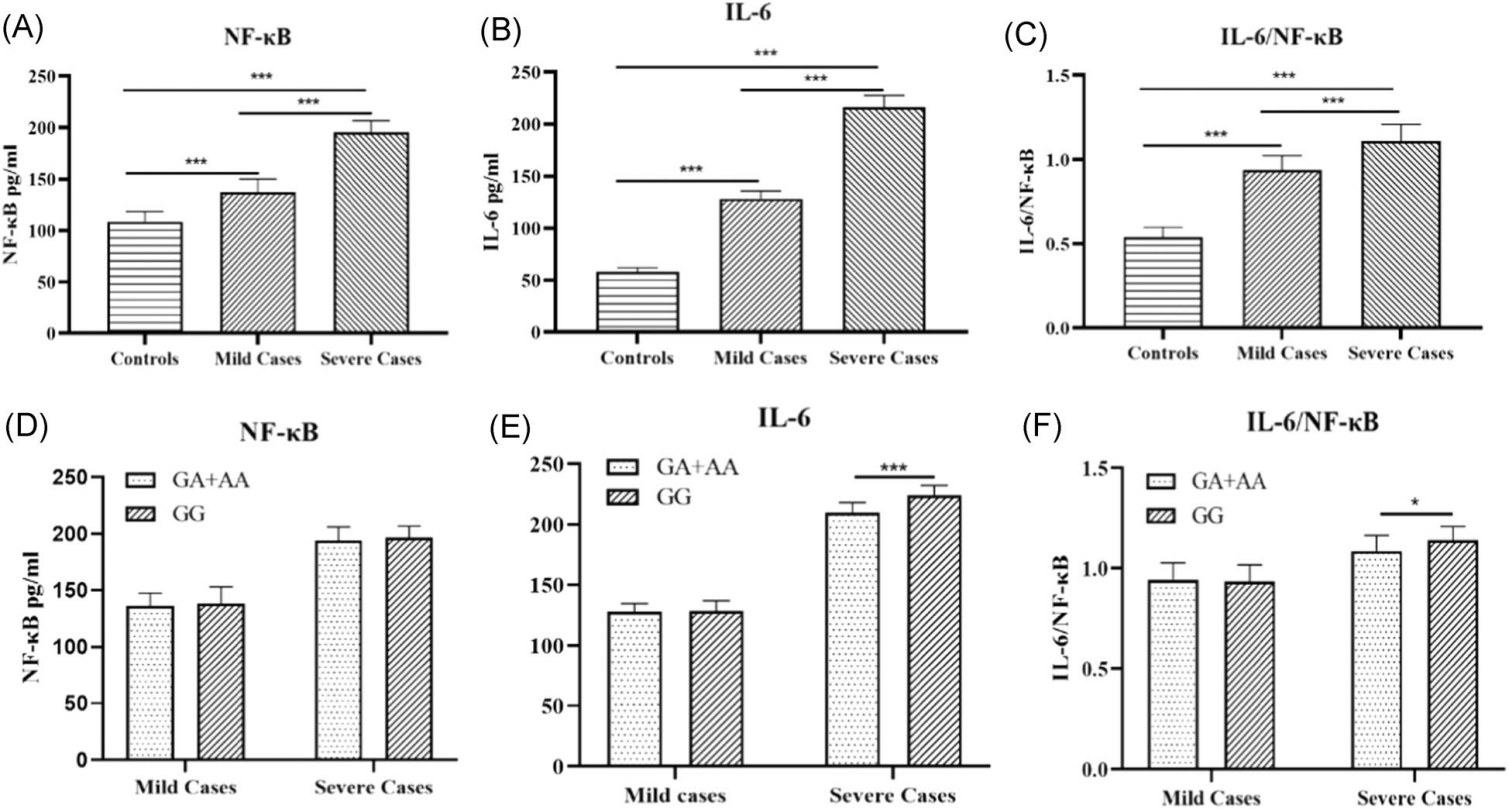
Serum NF‐κB and IL‐6 levels were measured in 32 controls, 31 mild EV71‐infected patients, 39 severe EV71‐infected patients. Values expressed as mean ± SD. (A) NF‐κB serum levels in mild EV71‐infected patients and severe cases were significantly higher than in controls (*p *< .001 and *p* < .01, respectively). The similar difference was found between mild and severe EV7‐infected cases (*p* < .001). (B) IL‐6 serum levels were significantly higher in mild and severe EV71‐infected patients than in controls (*p *< 0.001 and *p* < .001, respectively). The similar difference was found between mild and severe EV71‐infected cases (*p* < .001). (C) The IL‐6/NF‐κB ratios in mild and severe EV71‐infected cases were significantly higher than in controls (*p* < .001). The similar difference was found between mild and severe EV71‐infected cases (*p* < .001). (D) There were no significant differences between (GA + AA) and GG genotypes patients both in mild EV71‐infected cases and in severe EV71‐infected cases. (E) The serum levels of IL‐6 in (GA + AA) genotypes in severe EV71 patients were significantly lower than in GG genotypes patients (*p* < .001), but there was no difference in mild cases. (F) IL‐6/NF‐κB ratios in (GA + AA) genotypes in severe EV71 patients were significantly lower than in GG genotypes patients (*p* < .05), but there was no difference in mild cases. * p < .05, ***p < .001

Serum levels of NF‐κB and IL‐6 increased obviously in patients with mild (137.3 ± 13.0 pg/ml, *p* < .001 and 128.2 ± 7.7 pg/ml, *p* < .001) and severe EV71‐infection (195.3 ± 11.4 pg/ml, *p* < .001 and 216.4 ± 11.2 pg/ml, *p* < .001) compared to levels in uninfected children (108.7 ± 9.8 pg/ml and 58.2 ± 3.8 pg/ml) (Figure 3A, B). The ratio of IL‐6/NF‐κB was similar between patients with mild (0.9 ± 0.1, *p* < .001) and severe (1.1 ± 0.1, *p* < .001) EV71 infection (Figure 3C) when compared to that in the controls (0.5 ± 0.1). No obvious difference was found in the serum levels of NF‐κB in the various genotypes among the mild and severe EV71 cases (Figure 3D). In severely infected EV71 patients, the serum level of IL‐6 in the GA + AA genotype (209.8 ± 8.5 pg/ml) was significantly lower than that in the GG genotype (224.1 ± 8.3 pg/ml, *p* < .001), but there was no significant difference in mildly infected patients (Figure 3E). Furthermore, we found that the ratio of IL‐6/NF‐κB in the GA + AA genotype (1.1 ± 0.1) was obviously lower than that in the GG genotype (1.2 ± 0.1) in severe cases (*p* < .05) (Figure 3F).

## 4 DISCUSSION

EV71 infection, especially severe infection, can affect multiple organ functions and cause serious neurological complications, such as brainstem encephalitis, flaccid paralysis, and neurogenic pulmonary edema. In the past few decades, EV71 has caused several outbreaks in the Asia‐Pacific region, seriously threatening the health of children. Although an increasing number of epidemiological and biological studies have revealed the connection between genetic factors and EV71 infection, the exact pathological mechanisms remain unclear. Some polymorphisms of EV71 receptors (such as TLR3 and TLR4) have been shown to be involved in the inflammatory pathogenesis of EV71 infection.^6^, ^17^ Genetic polymorphisms of some inflammatory mediators (such as *OAS3, IL‐4*, and *CPT2*) have also been shown to be associated with susceptibility to EV71 infection.^5^, ^18^, ^19^, ^20^, ^21^ Evidence that host genetic polymorphisms affect the susceptibility and severity of EV71 infection is increasing. IRAK4 is indispensable for responses to IL‐1 and ligands that stimulate various TLRs; therefore, it plays a significant role in mediating the innate immune response induced by EV71. In this study, we explored the relationship between the polymorphism of rs4251545 in *IRAK4* and EV71 infection.

We found an obviously higher frequency of the GA + AA genotypes and A allele in EV71 infection cases, especially in severe cases, than in controls. This revealed that patients with the GA + AA genotype had increased susceptibility to EV71 infection, which was consistent with the results of hepatitis B virus‐(HBV)‐related HCC.^15^ We also found that the frequencies of the GA + AA genotypes and A allele in severely infected EV71 patients were distinctly higher than those in mild EV71 cases. However, among severe cases, there were no significant differences between EV71 cases with encephalitis and those without. These results suggest that the polymorphism of rs4251545 in *IRAK4* is involved in the pathogenesis of severe EV71 infection. Wang et al.^15^ pointed out that the A allele is positively associated with the risk of HBV‐related HCC because the A allele reduced *IRAK4* expression in the liver and increased the proliferation rate of L02 cells. Jun et al.^22^ also indicated that the rs4251545 A allele is associated with severe sepsis. These findings suggest that the A allele may be related to severe EV71 infection, but the pathological mechanism for this effect is not yet clear. In addition, we found that the mRNA expression of *IRAK4* in mildly and severely EV71‐infected children was obviously increased compared with that in the controls. Patients with GA + AA genotypes had higher mRNA expression of *IRAK4* than those with GG genotypes. This showed that the *IRAK4* rs4251545 A allele might affect the mRNA expression of *IRAK4* in patients with mild and severe EV71 infection.

According to the comparison of clinical manifestations and laboratory data of different genotypes, our study found no significant differences in gender and age between the GA + AA and GG genotypes. This result is inconsistent with those of Wang et al.^23^, ^24^ who showed that individuals aged <4 years old and males are prone to develop severe EV71 infection. This may be due to the different sample sizes and different regional distributions of research objects. In addition, we found that patients with the GA + AA genotype tended to have a longer duration of fever, higher incidence of seizure, higher incidence of spirit change, higher incidence of abnormal EEG, and higher WBC counts and BG concentrations than those with the GG genotype, but no differences were found in EV71 load, the incidence of abnormal brain MRI, and vomiting. This is consistent with the results of Chang et al.^23^ and Li et al.^24^ that WBC and BG are key indicators of severe EV71 infection. Prolonged fever duration, higher incidence of seizure, higher incidence of spirit change, higher incidence of abnormal EEG have been identified as obvious risk factors for encephalitis. These valuable clinical data can be used to determine the severity of EV71 infection in the early stages and help us take active intervention measures to prevent disease progression and reduce the incidence and mortality of severe EV71 infection. Overall, rs4251545 participates in the inflammatory process of EV71 infection, causing the corresponding pathological changes, but its pathogenesis needs further exploration.

NF‐κB is a transcription factor of the Rel family, which integrates multiple stress stimuli to modulate gene expression events during EV71 infection. A previous study^12^ has shown that IRAK4 and NF‐κB are involved in IL‐6 gene expression induced by Serum Amyloid A in dermal fibroblasts. IRAK4 mediates NF‐κB activation by affecting the transmission of information between TIR and MyD88, thus causing the release of downstream inflammatory factors, such as IL‐6 and IL‐1β. Therefore, we not only explored the expression of *IRAK4* mRNA but also selected NF‐κB and IL‐6 as the main indicators for further study. The IL‐6/NF‐κB ratio was significantly higher in severe EV71 cases than in the controls and mild cases. A similar discrepancy was found in the IL‐6 and NF‐κB serum levels. These results suggested that activation of NF‐κB and the release of IL‐6 may be risk factors for severe EV71 infection. This was consistent with previous experiments showing that macrophages, functioning not only as target cells but also as effectors, could change their expression of NF‐κB and IL‐6 when infected by EV71 in vitro.^25^ IL‐6 expression also reportedly increased gradually with the severity of EV71 infection. At the same time, some studies^26^ have shown that IL‐6 expression increased significantly within 48 h of disease aggravation, but not significantly in the later stage of the disease. For severe EV71 cases, serum IL‐6 was obviously lower in the GA + AA genotype group than in the GG genotype group, but there was no significant difference in mild cases. No difference in NF‐κB expression in different genotypes was found in severe cases. The data revealed that the A allele was prone to interfere with the production of IL‐6 and patients with this allele were more likely to suffer from severe EV71 infection. A previous study^15^ indicated that the plausibility of reduced proinflammatory and increased proliferation rate of cells could be attributed to the unbalanced immunity reaction induced by rs4251545 in HCC. IL‐6 levels and the ratio of IL‐6/NF‐κB were much higher in severe cases than in mild cases but were lower in GA + AA genotypes than in GG genotypes among severe cases. This is consistent with Wang's study^15^ that rs4251545 reduced the expression levels of proinflammatory cytokines, such as IL‐6, CXCL‐1, and IL‐8, mainly due to immune system dysfunction and the explosive inflammatory response caused by severe EV71 infection.

The exact mechanism by which IRAK4 rs4251545 affects the susceptibility and severity of EV71 infection is unclear, but the following postulation may be admitted. Regarding our results, we hypothesized that rs4251545 might alter the transcription process, thereby affecting the signal transmission of the TIR pathway and the expression of proinflammatory cytokines. More specifically, the rs4251545 locus occurred in an enhancer/silencer region of the gene and caused an alanine‐to‐threonine substitution at amino acid 428, which might impact the *IRAK4* transcription level. Combining the ELISA results of IL‐6/NF‐κB and qRT‐PCR results of *IRAK4*, we found that rs4251545 affected the production and release of downstream inflammatory factors and signaling molecules by affecting the expression level of *IRAK4*. Dysregulation of TLR signaling pathway activation due to changes in *IRAK4* expression might disrupt the balance between the production of pro‐ and anti‐inflammatory cytokines and would have profound effects on the susceptibility and severity of EV71 infection.^27^

This study had some limitations. First, the samples we collected were not sufficient to represent the whole population; therefore, the characteristics of the study sample only reflected a portion of the entire population. Second, other cytokines associated with the immune system in the serum were not detected and analyzed. Third, no genotyping and sequencing analysis of the EV71 virus was carried out in this study, but C4 was the most popular type in this period according to other reports.^28^, ^29^, ^30^ Moreover, the mechanism by which the *IRAK4* rs4251545 polymorphism affects the function of IRAK4 in EV71 infection progression should be elucidated.

## 5 CONCLUSION

The IRAK4 rs4251545 polymorphism was associated with the susceptibility and severity of EV71 infection. The A allele is a susceptibility factor for the development of severe EV71 infection in Chinese children.

## AUTHOR CONTRIBUTIONS

**Jie Song**: Data curation; writing—original draft; visualization, investigation. **Yedan Liu**: Data curation; methodology; software; writing—review and editing. **Ya Guo**: Validation; formal analysis; data curation; writing—review and editing. **Peipei Liu**: Validation; data curation; visualization. **Fei Li**: Validation; resources; data curation. **Chengqing Yang**: Validation; investigation; resources. **Xiaoyu Pan**: Validation; investigation; resources. **Liping Yi**: Validation; investigation; resources. **Fan Fan**: Validation; writing—review and editing. **Han Zhao**: Writing—review and editing. **Zongbo Chen**: Validation; writing—review and editing; supervision; project administration; funding acquisition. All authors contributed to the writing of the final manuscript. All members of the our Study Team contributed to the management or administration of the trial.

## CONFLICTS OF INTEREST

The authors declare no conflicts of interest.

## Data Availability

The data that support the findings of this study are available from the corresponding author upon reasonable request.
